# Impact of negative symptoms on health-related quality of life in schizophrenia

**DOI:** 10.3389/fpsyt.2023.1252354

**Published:** 2023-09-07

**Authors:** Yanhui Li, Gurpreet Rekhi, Mei San Ang, Jimmy Lee

**Affiliations:** ^1^East Region, Institute of Mental Health, Singapore, Singapore; ^2^Research Division, Institute of Mental Health, Singapore, Singapore; ^3^North Region and Department of Psychosis, Institute of Mental Health, Singapore, Singapore; ^4^Lee Kong Chian School of Medicine, Nanyang Technological University, Singapore, Singapore

**Keywords:** schizophrenia, negative symptoms, health-related quality of life, motivation and pleasure, asociality

## Abstract

Evidence regarding the association of Negative Symptoms (NS) dimensions with Health-related Quality of Life (HRQoL) is limited and no prior study has looked into contributions of NS domains on HRQoL. This study bridges the gap by examining the associations of NS, its two dimensions (Motivation and Pleasure, and Emotional Expressivity) and five domains (Anhedonia, Avolition, Asociality, Blunted affect and Alogia) with HRQoL in schizophrenia. 274 individuals with schizophrenia were assessed on the Positive and Negative Syndrome Scale (PANSS) and Brief Negative Symptom Scale (BNSS). PANSS scores were mapped to EuroQol five-dimensional (EQ-5D-5L) utility scores using an algorithm previously validated in Singapore, and the resulting EQ-5D-5L scores were used as a measure of HRQoL. Multiple linear regression analyses of the two NS dimensions and five NS domains against EQ-5D-5L showed that a lower severity of NS, specifically that of the Motivation and Pleasure (MAP) dimension and asociality domain was associated with higher HRQoL. Our findings highlight the importance of targeting NS, particularly MAP and asociality, in improving HRQoL in schizophrenia.

## Introduction

Quality of Life (QoL) is an important outcome in the treatment of diseases including mental illnesses. It is defined by the World Health Organization as “individuals’ perceptions of their position in life in the context of the culture and value systems in which they live, and in relation to their goals, expectations, standards, and concerns” (1). Broadly, QoL can be viewed as a construct consisting of four dimensions — general well-being (general satisfaction with life), objective QoL (observable social and material wellbeing), subjective QoL (individual satisfaction with social and material wellbeing) and Health-related Quality of Life (HRQoL) (2).

HRQoL measures the impact of chronic disease on an individual’s self-reported perception of his or her physical, emotional, mental, and functional well-being (3). HRQoL in psychiatry can be measured by three broad categories of instruments (4): generic instruments such as the EuroQol five-dimensional (EQ-5D) (5) and Short Form 36 Health Survey (SF-36) (6), severe mental illness HRQoL instruments such as the Quality of Life Enjoyment and Satisfaction Questionnaire (Q-LES-Q) (7), or disease-specific HRQoL instruments such as the Quality-of-life Questionnaire in Schizophrenia (S-QoL) (8). While there is ongoing debate on whether generic or specific instruments are preferred, regulatory bodies in the USA, UK and France have endorsed the use of generic instruments, i.e., EQ-5D or SF-36, in assessing treatment outcomes of chronic health conditions because it can be adopted for cross disease comparisons and is cost-effective (9, 10). Notably, generic instruments such as EQ-5D and SF-36 have been found to correlate well with the disease-specific Schizophrenia Quality of Life Scale (SQLS) (9).

Negative symptoms (NS) in schizophrenia consist of two dimensions - Motivation and Pleasure (MAP), and Emotional Expressivity (EE), and five symptom domains (Anhedonia, Avolition, Asociality under MAP; Blunted affect and Alogia under EE) (11). Negative symptoms contribute significant burden in schizophrenia and have consistently been associated with lower QoL in schizophrenia patients (12, 13). While studies have adopted different measures, no specific HRQoL instrument has been validated for use in negative symptoms (14). Evidence regarding association of NS dimensions with HRQoL is limited, with a few studies previously suggesting a negative association between MAP and HRQoL (15, 16). However, no prior study has looked into contributions of the five NS domains on HRQoL (16).

The five-level version of the EuroQol five-dimensional (EQ-5D-5L) has been found to have good construct validity in schizophrenia (17, 18), and is sensitive to differences in HRQoL even among varying degrees of illness severity in schizophrenia (17). Unfortunately, the EQ-5D-5L is not always available due to logistical limitations. In the absence of generic preference-based instruments, estimates of HRQoL are obtained at times by mapping clinical symptomatology scales to HRQoL measures. This approach has been adopted and validated by multiple previous studies across a range of diseases, including mental illnesses (19, 20). In the present study, we have adopted this approach and employed a validated algorithm (21) to map Positive and Negative Syndrome Scale (PANSS) scores (22) onto EQ-5D-5L, to obtain a single EQ-5D-5L utility score as an estimate of HRQoL. We examined the association between NS, its two dimensions and five domains on HRQoL in schizophrenia. We hypothesized higher severity of NS, in particular MAP, to be associated with a lower HRQoL.

## Materials and methods

### Subjects

Two hundred and seventy-seven participants with a diagnosis of schizophrenia, aged 21 to 65 years were recruited from outpatient clinics at the Institute of Mental Health, Singapore. Those with a current alcohol or substance use disorder, intellectual disability, or a history of head injury or neurological disorder were excluded. Three participants withdrew from the study and did not complete the clinical assessments, hence sample reported for this study is 274. Written informed consent was obtained from all participants. Details on antipsychotic prescriptions were collected from existing medical records. Daily antipsychotic doses were converted into chlorpromazine equivalents (CPZeq) (23, 24).

### Study assessments

All participants were assessed on the Positive and Negative Syndrome Scale (PANSS) (22). PANSS scores were mapped to EQ-5D-5L utility scores via an algorithm previously validated in Singapore, based on a sample of 239 patients aged 21 years and above with a diagnosis of schizophrenia spectrum disorders recruited from outpatient clinics of the same psychiatric hospital (21). The authors had used Ordinary Least Squares (OLS), censored least absolute deviations and Tobit regression methods to estimate EQ-5D-5L utility scores and had identified a best-fit model out of 18 regression models. This model explained up to 33.8% of the variation with minimal mean square error (0.0328) and mean absolute error (0.1348) in the study (21). PANSS positive, negative, general psychopathology scores, age and gender were used to derive EQ-5D-5L utility scores via the following algorithm:

EQ-5D-5L utility = 1.3103–0.0044 × positive + 0.0025 × negative – 0.0146 × general psychopathology – 0.0029 × age + 0.0149 × female.

EQ-5D-5L utility scores calculated were used as an estimate of HRQoL in subsequent analyses.

NS were assessed using the Brief Negative Symptom Scale (BNSS) consisting of 13 items; each item rated from 0 (normal) to 6 (extremely severe) (25). BNSS MAP-EE dimension and five BNSS domain scores were computed using summation method. Negative symptom remission (NSR) was assessed using scores on the five BNSS domains based on predetermined criteria (26). The Calgary Depression Scale for Schizophrenia (CDSS) (27) was used to assess severity of depressive symptoms.

### Statistical analyses

All statistical analyses were carried out using IBM SPSS Statistics Version 29.0. Association between EQ-5D-5L and NS was examined with EQ-5D-5L as the dependent variable and BNSS Total, MAP and EE, BNSS five domains and NSR as independent variables in four separate multiple linear regression models. Based on previous reported associations, the effects of age, sex, highest education level, body mass index, marital status, employment status, age at onset of psychotic symptoms and severity of depressive symptoms on EQ-5D-5L were controlled for in all models (28–37). Minimum sample size required based on power analysis for a power of 0.80 (alpha = 0.05) would be 138 for a model with 15 predictor variables, assuming medium effect size (38). Our study has an effective sample size of 274 and is sufficiently powered to obtain reliable results from the regression analyses with 13 independent variables. Categorical data were coded as follows: sex (males 0, female 1); marital status (unmarried 0, married 1), employment status (unemployed 0, employed 1).

## Results

Table 1 shows demographics and clinical characteristics of the study sample. The sample consisted of 152 (55.5%) males and 122 females (44.5%), with a mean age of 40.42 (*SD* = 10.17, range = 21 to 65) years, and a mean BMI of 26.32 (*SD* = 5.09) kg/m^2^. Majority of participants were of Chinese ethnicity (*n* = 231, 84.3%). Thirty-five (12.8%) participants were married. Highest educational level was as follows: Primary & below, *n* = 44 (16.1%), Secondary, *n* = 60 (21.9%), Pre-U, *n* = 20 (7.3%), Certificate or Vocational, *n* = 50 (18.2%), Diploma, *n* = 71 (25.9%), Degree and above, *n* = 29 (10.6%). Approximately half of them (*n* = 126, 46.0%) were employed.

**Table 1:** Demographics and clinical characteristics of the study sample.

Variable	*N* (%)
Sex – Males (%)	152 (55.5)
Ethnicity	
Chinese (%)	231 (84.3)
Indian (%)	22 (8.0)
Malay (%)	20 (7.3)
Others (%)	1 (0.4)
Marital Status (Married, %)	35 (12.8)
Highest educational level	
Primary and below (%)	44 (16.1)
Secondary (%)	60 (21.9)
Pre-University (%)	20 (7.3)
Certificate or Vocational (%)	50 (18.2)
Diploma (%)	71 (25.9)
Degree and above (%)	29 (10.6)
Employment status (Employed, %)	126 (46.0)
Antipsychotic type	
None (%)	4 (1.5)
Typical antipsychotics (%)	55 (20.1)
Atypical antipsychotics (%)	131 (47.8)
Both typical and atypical antipsychotics (%)	84 (30.7)
Negative symptom remission (NSR, %)	89 (32.5)
	Mean (S.D.)
Age (years)	40.42 (10.17)
BMI (kg/m^2^)	26.32 (5.09)
Age at onset of psychotic symptoms (years)	23.08 (6.51)
Duration of illness (years)	17.34 (9.64)
Antipsychotic treatment	
Duration of Antipsychotic Treatment (years)	15.05 (9.36)
Antipsychotic Dose (mg in CPZeq*)	462.35 (407.65)
CDSS Total score	2.84 (3.18)
BNSS Total score	24.92 (11.88)
BNSS MAP score	14.50 (6.35)
BNSS EE score	9.53 (7.13)
BNSS Anhedonia score	7.09 (3.48)
BNSS Asociality score	2.82 (1.79)
BNSS Avolition score	4.58 (2.17)
BNSS Blunted Affect score	5.43 (4.54)
BNSS Alogia score	4.10 (3.53)
PANSS Total score	58.11 (12.88)
PANSS Positive score	12.95 (4.92)
PANSS Negative score	17.72 (5.58)
PANSS General Psychopathology score	27.44 (5.96)
EQ-5D-5L score	0.79 (0.10)

*Antipsychotic doses were converted into daily chlorpromazine equivalents (CPZeq).

Mean age at onset of psychotic symptoms was 23.08 (*SD* = 6.51) years and mean duration of illness was 17.34 years (*SD* = 9.64). 54.4% (*n* = 149) of subjects were on antipsychotic monotherapy. 20.1% (55) of subjects were on typical antipsychotics only while 47.8% (131) of subjects were on atypical antipsychotics only. A total of 30.7% (84) of subjects were on both typical and atypical antipsychotics and 4 participants were not on any antipsychotics. Mean duration of antipsychotic treatment was 15.05 years (*SD* = 9.36, range = 0.46 to 43.82), while mean antipsychotic dose in daily chlorpromazine equivalents (CPZeq) was 462.35 mg (*SD* = 407.65).

Mean total CDSS score was 2.84 (*SD* = 3.18). Mean PANSS total score was 58.11 (*SD* = 12.88), corresponding to the “mild” severity (score of 55–62) on the Clinical Global Impression Severity rating scale (39). EQ-5D-5L scores ranged from 0.48 to 0.96, with a mean of 0.79 (*SD* = 0.10). Scores on BNSS were as follows: BNSS total, *M* = 24.92 (*SD* = 11.88), MAP, *M* = 14.50 (*SD* = 6.35), EE, *M* = 9.53 (*SD* = 7.13), Anhedonia, *M* = 7.09 (*SD* = 3.48), Asociality, *M* = 2.82 (*SD* = 1.79), Avolition, *M* = 4.58 (*SD* = 2.17), Blunted affect, *M* = 5.43 (*SD* = 4.54) and Alogia, *M* = 4.10 (*SD* = 3.53). Eighty-nine (32.50%) participants were in NSR.

Lower scores on BNSS total (β = −0.115, *t* = −2.149, *p* = 0.033), BNSS MAP (β = −0.127, *t* = −2.133, *p* = 0.034), and BNSS Asociality (β = −0.143, *t* = −2.279, *p* = 0.023) were significantly associated with higher EQ-5D-5L scores (see Table 2). Individuals in NSR were more likely to have higher EQ-5D-5L scores (*M* = 0.82, *SD* = 0.09) than those not in remission (*M* = 0.77, *SD* = 0.10; β = 0.126, *t* = 2.341, *p* = 0.020). Higher age, lower age at onset of psychotic symptoms and higher severity of depressive symptoms as assessed by CDSS were also significantly associated with lower HRQoL, as would be expected.

**Table 2:** Multiple linear regressions with BNSS scores and covariates on EQ-5D-5L.

	Model 1					Model 2					Model 3				
	β	*t*	*p*	Toler-ance	VIF	β	*t*	*p*	Toler-ance	VIF	β	*t*	*p*	Toler-ance	VIF
Age	−0.333	−5.862	**<0.001**	0.776	1.289	−0.325	−5.682	**<0.001**	0.758	1.318	−0.320	−5.588	**<0.001**	0.749	1.335
Sex	0.003	0.053	0.958	0.947	1.056	0.001	0.013	0.990	0.948	1.055	0.015	0.281	0.779	0.921	1.086
Highest education level	0.011	0.221	0.825	0.950	1.053	0.003	0.051	0.959	0.936	1.068	−0.014	−0.269	0.788	0.916	1.092
Body mass index	0.011	0.223	0.824	0.960	1.041	0.005	0.097	0.923	0.953	1.050	−0.017	−0.320	0.749	0.902	1.109
Marital status	0.065	1.237	0.217	0.894	1.119	0.059	1.113	0.267	0.888	1.126	0.042	0.786	0.433	0.865	1.156
Employment status	0.016	0.300	0.765	0.912	1.096	0.002	0.030	0.976	0.884	1.131	0.016	0.282	0.778	0.776	1.288
Age at onset of psychotic symptoms	0.111	1.985	**0.048**	0.806	1.241	0.114	2.050	**0.041**	0.805	1.243	0.116	2.083	**0.038**	0.789	1.267
Antipsychotic dose (in CPZeq)	−0.041	−0.786	0.433	0.908	1.101	−0.041	−0.779	0.437	0.909	1.101	−0.042	−0.801	0.424	0.896	1.116
CDSS total	−0.496	−9.655	**<0.001**	0.949	1.054	−0.479	−9.128	**<0.001**	0.902	1.109	−0.503	−9.374	**<0.001**	0.854	1.171
BNSS total	−0.115	−2.149	**0.033**	0.871	1.148	-	-	-	-	-	-	-	-	-	-
BNSS MAP	-	-	-	-		−0.127	−2.133	**0.034**	0.699	1.431	-	-	-	-	-
BNSS EE	-	-	-	-	-	−0.043	−0.784	0.434	0.831	1.204	-	-	-	-	-
BNSS Anhedonia	-	-	-	-	-	-	-	-	-	-	0.052	0.682	0.496	0.430	2.324
BNSS Asociality	-	-	-	-	-	-	-	-	-	-	−0.143	−2.279	**0.023**	0.625	1.601
BNSS Avolition	-	-	-	-	-	-	-	-	-	-	−0.070	−0.909	0.364	0.420	2.382
BNSS Blunted Affect	-	-	-	-	-	-	-	-	-	-	0.036	0.582	0.561	0.645	1.551
BNSS Alogia	-	-	-	-	-	-	-	-	-	-	−0.095	−1.436	0.152	0.566	1.766

Model parameters: Model 1: R^2^ = 0.343, Adj. R^2^ = 0.318, *F*(10,263) = 13.710, *p* < 0.001. Model 2: R^2^ = 0.349, Adj. R^2^ = 0.322, *F*(11,262) = 12.768, *p* < 0.001. Model 3: R^2^ = 0.363, Adj. R^2^ = 0.329, *F*(14,259) = 10.560, *p* < 0.001.*p* values < 0.05 are bolded.

## Discussion

As hypothesized, our study showed that a higher severity of negative symptoms, particularly in the MAP dimension and asociality domain, was significantly associated with lower HRQoL in schizophrenia, after controlling for covariates. Those in NSR had higher HRQoL than those not in NSR.

Savill et al. (16) previously studied the impact of negative symptoms on subjective quality of life (SQOL) in schizophrenia and found SQOL to be primarily related to experiential deficits, as opposed to expressive deficits (16). This is consistent with what we found, although we looked at HRQoL rather than SQOL. While we did not find a significant relationship between EE and QoL, García *et al* (2022) found both MAP and EE assessed through Clinical Assessment Interview for Negative Symptoms (CAINS) to be negatively associated with QoL assessed on the Quality of Life Scale (QLS) (15). Nonetheless, MAP was reported to have a stronger association with QoL when compared to EE (15).

Interestingly, our study found that the severity of asociality was negatively associated with HRQoL in schizophrenia. We could not find any previous study to compare these results. However, better social network was previously found to be associated with higher SQOL in schizophrenia (40). Psychosocial factors such as social rejection and social isolation have also been found to be major determinants for schizophrenia-specific QoL, as compared to sociodemographic, psychopathological, and neurocognitive factors (41). We postulate that asociality is associated with reduced protective effect of social factors against other factors such as psychopathological factors and their resulting distress, in accordance with Ritsner’s distress model of the HRQoL (42).

The significant association of asociality with HRQoL has important clinical implications. Asociality has been shown to be related to both disease and lifestyle factors such as cohabitation and involvement in vocational activities (43). Addressing factors related to asociality might help to decrease severity of asociality and in turn improve HRQoL. Some therapeutic modalities might include improving social cognition (44), or cognitive behavioural therapy to identify automatic thoughts contributing to asociality (45).

To our knowledge, this is the first study examining associations between the five domains of negative symptoms and HRQoL in schizophrenia. Our study has some limitations. Firstly, EQ-5D-5L scores were derived from PANSS scores, so one may argue the findings reflected the association between negative symptom domains of PANSS and negative symptoms measured by BNSS. However, the PANSS and BNSS are significantly different in their assessment of NS. PANSS has good measures for blunted affect and alogia, but is limited in its assessment of avolition, asociality, and anhedonia (46). On the other hand, BNSS assesses the five domains of NS comprehensively, including internal motivation and pleasure related to activities. Additionally, correlation between EQ-5D-5L scores and BNSS Asociality remained significant with only a small drop in strength after partialling out the effect of PANSS item N4 (Passive/Apathetic Social Withdrawal), the main item assessing asociality in PANSS (see Supplementary Table 1). This suggests results of BNSS Asociality are unlikely to be driven by the relationship with PANSS-derived EQ-5D-5L scores and the PANSS Asociality item. Generic preference-based measures are not always available in clinical practice, and many have turned to mapping as an alternative solution (47, 48). In the absence of available data on direct measurements of HRQoL, the PANSS-derived EQ-5D-5L scores are a reasonable estimate. Future research using direct HRQoL measurements would be ideal to compare with our findings. Although the algorithm by Abdin et al. (21) that we have employed has yet to be replicated in other studies, our study population was recruited from the same psychiatric hospital in Singapore and patient demographics in terms of age, gender and ethnicity (Table 1) were similar to the sample used in derivation of the above algorithm. Hence, we believe it is valid to employ the algorithm in mapping PANSS scores to EQ-5D-5L utility scores in this study. We also acknowledge that the effect of positive and general symptoms on HRQoL could not be controlled for in the regression analyses, and our study does not provide information on different domains of HRQoL. Lastly, our study population consisted of outpatients with mild to moderate severity of illness, so our results may not be generalisable to those with severe illness. Negative symptoms have been found to be more strongly correlated with lower QoL in stable schizophrenia as compared to schizophrenia in acute exacerbation (49), and also more strongly correlated with lower QoL in outpatients compared to inpatients (50). More research is needed to verify our findings in inpatient subjects with a higher severity of illness.

In conclusion, our study found that severity of negative symptoms, particularly in the MAP dimension and asociality domain, was negatively associated with HRQoL in individuals with schizophrenia. This study emphasizes the importance of addressing negative symptoms, in both research and clinical therapeutics, to improve outcomes in schizophrenia. To this, it is recommended that the management of negative symptoms adhere to evidence-based guidelines (51). Our study provides preliminary insights into the associations of the two dimensions and five domains of negative symptoms with HRQoL, and further research with other HRQoL measures is needed to support our findings.

## Data availability statement

The participants of this study did not agree for their data to be shared publicly, so supporting data is not publicly available. Further enquiries could be directed to either the corresponding author or to imhresearch@imh.com.sg.

## Ethics statement

The studies involving humans were approved by National Healthcare Group’s Domain-Specific Review Board (Ref: 2013/00996). The studies were conducted in accordance with the local legislation and institutional requirements. The participants provided their written informed consent to participate in this study.

## Author contributions

GR and JL designed the original study and wrote the protocol. GR and MA conducted data collection. YL and GR conducted literature review, data analyses, and wrote the first draft of the manuscript. MA and JL gave substantial comments and edited the manuscript. All authors contributed to the article and approved the submitted version.

## Funding

This study was supported by the Singapore Ministry of Health’s National Medical Research Council under the Centre Grant Programme (Grant No.: NMRC/CG/004/2013).

## Conflict of interest

JL has received honoraria from Otsuka, Janssen, Lundbeck and Sumitomo Pharmaceuticals.

The remaining authors declare that the research was conducted in the absence of any commercial or financial relationships that could be construed as a potential conflict of interest.

## Publisher’s note

All claims expressed in this article are solely those of the authors and do not necessarily represent those of their affiliated organizations, or those of the publisher, the editors and the reviewers. Any product that may be evaluated in this article, or claim that may be made by its manufacturer, is not guaranteed or endorsed by the publisher.
