# Supplementary material for: Impact of negative symptoms on health-related quality of life in schizophrenia
Source: Front Psychiatry. 2023 Sep 7;14:1252354. doi: 10.3389/fpsyt.2023.1252354 (PMC10512711; doi:10.3389/fpsyt.2023.1252354)
Supplement: Supplementary file 1 [file Table_1.DOCX]

**Supplementary Table 1.** Pearson correlations of EQ-5D-5L scores against BNSS MAP and Asociality scores, before and after controlling for PANSS N4 Asociality Item

|  | Correlation with EQ-5D-5L | | Partial correlation with EQ-5D-5L (controlling for PANSS N4 Asociality) | |
| --- | --- | --- | --- | --- |
|  | r | *p* | r | *p* |
| BNSS MAP | -0.213 | <0.01 | -0.128 | 0.035 |
| BNSS Asociality | -0.245 | <0.01 | -0.165 | 0.006 |
